# Supplementary material for: The causal role of circulating inflammatory markers in osteoporosis: a bidirectional Mendelian randomized study
Source: Front Immunol. 2024 Jul 18;15:1412298. doi: 10.3389/fimmu.2024.1412298 (PMC11291241; doi:10.3389/fimmu.2024.1412298)
Supplement: Supplementary file 1 [file DataSheet_1.zip › supplementary material/CIM-OSTEOPOROSIS /GCST90274785.csv_leave_one_out.pdf]

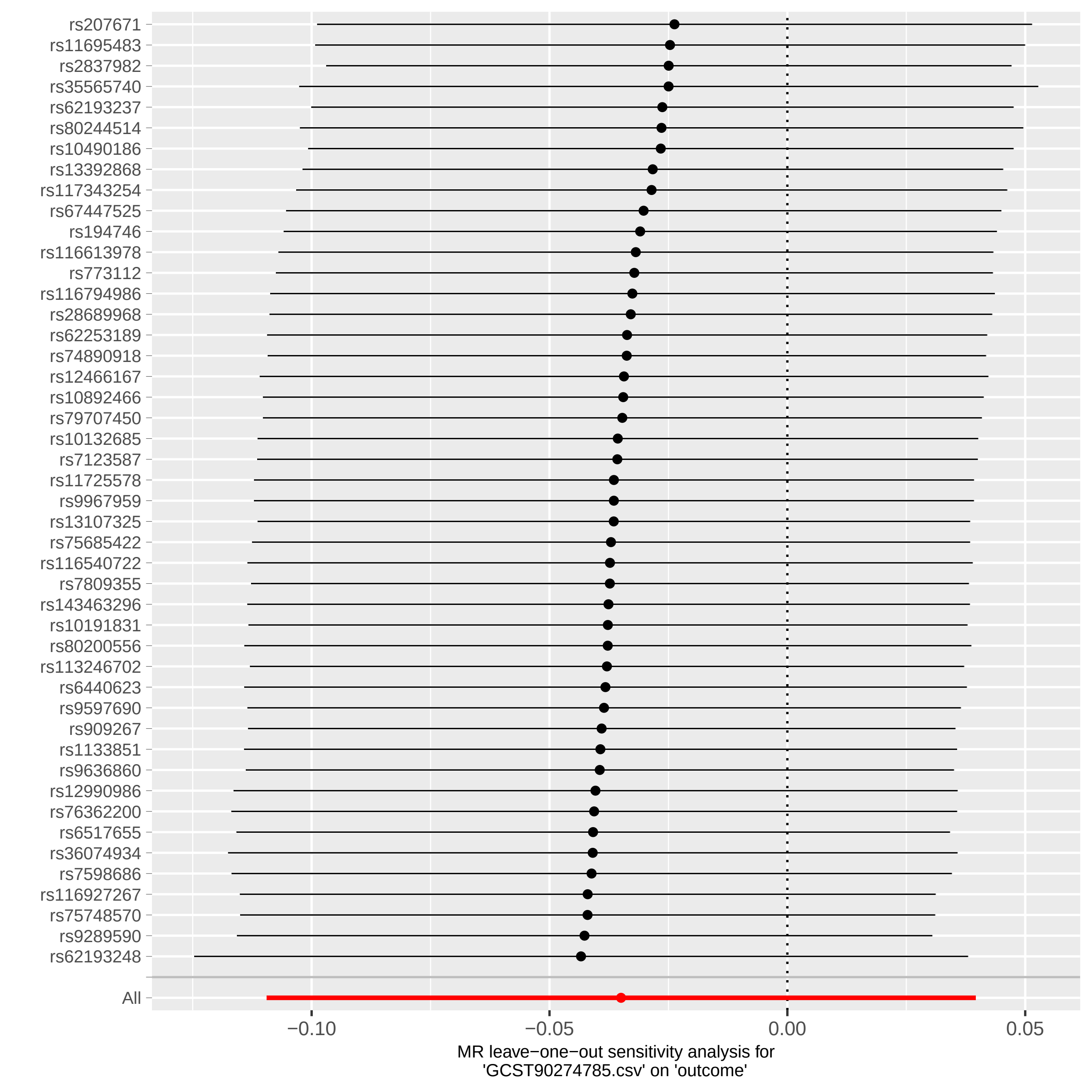
rs207671
rs11695483
rs2837982
rs35565740
rs62193237
rs80244514
rs10490186
rs13392868
rs117343254
rs67447525
rs194746
rs116613978
rs773112
rs116794986
rs28689968
rs62253189
rs74890918
rs12466167
rs10892466
rs79707450
rs10132685
rs7123587
rs11725578
rs9967959
rs13107325
rs75685422
rs116540722
rs7809355
rs143463296
rs10191831
rs80200556
rs113246702
rs6440623
rs9597690
rs909267
rs1133851
rs9636860
rs12990986
rs76362200
rs6517655
rs36074934
rs7598686
rs116927267
rs75748570
rs9289590
rs62193248
All
−0.10
−0.05
0.00
0.05
MR leave−one−out sensitivity analysis for
'GCST90274785.csv' on 'outcome'
